# High-Resolution EEG Amplifiers Are Feasible for Electrocochleography Without Time Restriction

**DOI:** 10.3390/audiolres15010008

**Published:** 2025-01-21

**Authors:** Florian Josef Schertenleib, Sabine Hochmuth, Jana Annina Müller, Pascale Sandmann, Andreas Radeloff

**Affiliations:** 1 Department of Otorhinolaryngology at Evangelisches Krankenhaus Oldenburg, University of Oldenburg, 26122 Oldenburg, Germany; sabine.hochmuth@uni-oldenburg.de (S.H.); andreas.radeloff@uni-oldenburg.de (A.R.); 2 Cluster of Excellence ‘Hearing4all’, University of Oldenburg, 26111 Oldenburg, Germany; 3 Research Center Neurosensory Science, University of Oldenburg, 26129 Oldenburg, Germany

**Keywords:** electrocochleography, speech decoding, human auditory system, high-resolution recording

## Abstract

Objectives: The gold standard for electrocochleography (ECochG) is using dedicated recording devices for auditory evoked potentials. However, these have a very limited time window for recording. The aim of this study is to evaluate EEG amplifiers for ECochG, in particular for recording cochlear microphonics (CMs) without time restriction. Methods: Three high-resolution EEG amplifiers and different types of electrodes were analyzed and compared with a clinical system for recording auditory evoked potentials. For this, CMs were recorded after stimulation with various stimuli in a dummy and in human subjects. In the latter, recordings were made from the tympanic membrane and, during otosurgical procedures, from the promontory. Our evaluation focused on comparing signal amplifiers and electrode types, considering the signal-to-noise ratio, recording characteristics, and measurement reliability. Results: Using a dummy model, we observed significant differences among devices, electrode types, and stimulus frequencies. These findings were subsequently confirmed in human participant measurements. Nevertheless, EEG amplifiers proved to be feasible for ECochG recordings and offered a recording fidelity comparable to proprietary clinical methods. Importantly, with EEG amplifiers, we were able to record cochlear potentials in response to speech stimuli, revealing a strong correlation (r = 0.78) between recorded signals and the input stimulus. Conclusions: Our findings indicate that high resolution EEG amplifiers are suitable for recording cochlear potentials, in particular, CMs. This allows for evaluating cochlear signals in response to extended stimuli, in particular, speech stimuli.

## 1. Introduction

Electrocochleography (ECochG) refers to the recording of electrical potentials originating from the cochlea. This includes cellular signals from the hair cells and early neural potentials from the spiral ganglion cells forming the auditory nerve. Frequency-specific depolarization of hair cells in the cochlea generates an electrical field that can spread through tissue [1,2,3,4]. ECochG signals are best detected near their origin, e.g., inside the cochlea via cochlear implants [5], at the round window membrane or at the stapes footplate (4). However, it can still be measured using electrodes placed onto the tympanic membrane or in the outer ear canal [6,7,8,9,10,11,12].

The ECochG signal includes several stimulus-related potentials, among them the cochlear microphonics (CMs) that arise predominantly from the outer hair cells. The CM mirrors the acoustic waveform with minimal latency and is phase-correlated with the stimulus. First described by Wever and Bray [13], CMs have been studied for many years (for review, please see Eggermont [14]).

Electrocochleography has also been widely used in the clinical context [15,16,17,18,19,20]. Recent studies have focused on using ECochG to monitor residual hearing during cochlear implant (CI) surgery [4,21,22,23]. ECochG has also been used as a diagnostic tool [24], particularly with regard to auditory synaptopathy/neuropathy (ASAN [25] and Morbus Menière [8,26]).

CMs may also provide insights into efferent pathways of the auditory system that are believed to play a role in speech perception in noise [27]. Currently available devices for measuring ECochG in humans are built for measuring auditory evoked potentials (AEPs) that are in a time window of less than 1 s after stimulus presentation. Thus, these devices are not capable of recording longer time periods. This prevents the use in research questions that investigate complex stimuli, such as speech in noise, or include permanent measurements for correlation analyses.

To address these limitations, we propose a test setup using signal amplifiers typically employed in electroencephalography (EEG) and electrocorticography (ECoG). For this, several EEG amplifiers have been evaluated in vitro and in vivo. Additionally, the application of specific electrodes for ECochG and speech stimuli have been tested. In a limited sample, the usability of the proposed system in intraoperative invasive measurements and for a speech stimulus was also tested.

## 2. Materials and Methods

### 2.1. Participants

For the in vitro measurements, we utilized watermelons as a spherical dummy model. For the in vivo *non-invasive* measurements, seven healthy volunteers (2 males, 5 females; age range 24–32 years) with normal hearing were recruited from the University of Oldenburg. Participants underwent otologic examination, tympanometry, and pure tone audiometry (AT1000, AURITEC, Hamburg, Germany), which confirmed normal hearing and normal middle-ear function. According to WHO criteria, normal hearing indicates hearing thresholds of ≤20 dB HL across the frequency range of 250–8000 Hz, with no clinical pathologies affecting sound conduction (middle ear) or sound perception (inner ear). For the in vivo *invasive* measurements, eight hearing-impaired adult patients (6 males, 2 females; age range 21–69 years), who were scheduled for ear surgery, were recruited. This study was approved by the local authorities responsible (Medizinische Ethikkommision).

### 2.2. Devices and Electrophysiological Measurements

We used an Eclipse AEP system for clinical measurements of the auditory system (Interacoustics, Middelfart, Denmark) with a 30 kHz maximum sampling rate and a 15 ms window length as a reference. Then, we assessed alternative amplifiers, including a neurosurgical monitoring system (ISIS, Inomed, Austria) with a maximum sampling rate of 20 kHz, an active EEG amplifier system (ActiveTwo, Biosemi, Amsterdam, The Netherlands) with a 16 kHz maximum sampling rate and a biosignal amplifier (g.USBAMP, gtec, Schiedlberg, Austria) with a 38.4 kHz maximum sampling rate. See Table 1 for details.

To capture external trigger information with, e.g., the ISIS device, we built customized connectors and a trigger box. This battery-operated digital-to-analog converter (DAC) provided a 5V TTL trigger pulse as output for seamless integration with external sound interface stimulation. The device is optically connected to ensure galvanic separation between the stimulation and recording devices and can be configured in MATLAB to interface with various amplifier systems. For additional details, including the circuit diagram of the trigger box, please refer to the Supplementary Materials.

For non-invasive recordings, we used particular tympanic membrane electrodes (TMtrodes, Sanibel Supply, Eden Prairie, MN, USA), placed via the ear canal at the tympanic membrane [7]. Invasive recordings were obtained by inserting a needle electrode (ECochG electrode, inomed Medizintechnik GmbH, Emmendingen, Germany) near the round window niche during anesthesia [4,11,22,28,29,30]. We used both sharp-tipped and blunt-tipped needle electrodes for comparison purposes. For the in vivo measurements, we placed the ground and inverting electrodes on the forehead (Fz), following the AEP instruction from Coraci and Beynon [6]. In the in vitro dummy model measurements, we approximated this setup on the melon surface, placing the ground and reference electrodes in analogous positions, and positioned the measurement electrode at the temporal pole (T4). This configuration does not represent a monopolar setup, as it includes the temporal electrode to provide more comprehensive signal capture.

To evaluate potential electromagnetic interference from the earphone transducer or wiring, we implemented a negative control condition, by clamping the air tubes of the earphone transducers. Interference could also be identified by a low latency between stimulus onset and response due to the instant transmission of the electric stimulus compared to the speed of sound though the connecting tube. To increase discriminatory power, we doubled the length of the sound tubes to enhance the latency effect.

### 2.3. Dummy Model

To simulate cochlear potentials in a controlled dummy model, we utilized a watermelon as a spherical model, adapting techniques described in Hofner, Albrecht [31]. For generating the electrical fields, an external sound card (Fireface UC, RME, Haimhausen, Germany) was utilized. We first cleaned the melon’s surface with alcohol pads and then inserted a dipole source, made from two twisted copper wires (Ø 1.5 mm), to create an electrical field. We employed the same types of electrodes as used in in vivo measurements, specifically attaching surface electrodes and two types of needle electrodes (sharp and blunt), mirroring the AEP setup with the ground electrode at Fpz, the inverting electrode at Fz, and the non-inverting electrodes (either surface or needle) positioned on one temporal pole (as depicted in the Supplementary Materials).

After preparing the melon, we connected the bare ends of an audio cable from the sound card to the wires of the dipole to apply an electric sound signal to the model. Before recording, electrode impedances were measured to confirm the correct electrode placement, and a sound clamp condition recording was conducted to check for interference by disconnecting the peripheral part of the dipole cable, analogous to clipping the air tubes of the earphone transducers during human measurements. Following this, the dipole cables were reconnected, and responses to test stimuli at various frequencies were measured multiple times with each of the four recording devices using both the surface and needle electrodes.

### 2.4. Human Electrocochleography

For non-invasive in vivo measurements, participants were instructed to lie down and relax on a bench inside an acoustically isolated room, remain still throughout the recording process, and keep their eyes closed. Invasive in vivo measurements were performed in an operating room with patients under general anesthesia. The subject’s skin was cleaned with alcohol pads and an abrasive gel. Surface electrodes and either TMtrodes or needle electrodes were then placed at their respective designated areas. The foam tip earphone was inserted into the external auditory canal of the ear to be measured, also serving to secure the TMtrode or the needle electrode in place. During the recordings, acoustic stimuli were presented through the earphones. Sound clamp conditions ensured that there was no interference from the stimulation device or the earphone transducers.

When recording with the ISIS device, one of the EMG channels had to be used for triggering. This modification deactivated the galvanic isolation, which represents an unacceptable safety risk for use on humans, so that it could only be used for recordings of dummy models.

### 2.5. Tympanic Membrane ECochG

To reduce electrical resistance on the skin surface in the ear canal, the TMtrode was soaked in saline for 10 min, and the ear was rinsed with saline for approximately 10 s prior to insertion. The electrode was then placed directly on the eardrum under microscopic vision to prevent injury as well as to control the position of the electrode in the lower half quadrants of the tympanic membrane [7]. The impedance of surface electrodes and TMtrodes was measured using the Eclipse AEP system and maintained below 20 kOhm. Surface electrodes typically showed values below 10 kOhm, while TMtrodes ranged between 10 and 20 kOhm.

### 2.6. Trans-Tympanic ECochG

The reference electrodes were attached to the cleaned skin surface. Then, the needle electrode was inserted under microscopic vision through the ear canal and eardrum, placed on the promontory of the cochlea in the middle ear, and secured with the earphone foam tip. We performed repeated sound clamp conditions and adjusted the components until electrical interference was eliminated. The reference Eclipse AEP system was used initially to monitor and optimize the needle electrode positioning for a clear response signal before proceeding with the protocol. Sharp needle electrodes presented impedance levels around 100 kOhm, consistent in both the dummy model and intraoperative measurements, which were beyond the measurement range of the Eclipse device. For these, impedance was verified using the g.USBAMP device. Blunt needle electrodes exhibited lower impedance values, around 30 kOhm, also consistent across the dummy model and intraoperative measurements. Further details on the electrodes and their placement can be found in the Supplementary Materials.

### 2.7. Stimuli and Stimulus Presentation

All stimuli of in vivo measurements were presented through insert earphones (E-A-RTONE, 3M, Maplewood, MN, USA). We used pure tone bursts (500, 1000, 2000, 4000 Hz) and a speech sample for our measurements. Tone bursts, with a duration of 10 ms including 1 cycle of ramping and a variable number of cycle plateaus depending on the stimulus frequency, were presented at a rate of 57.8 per second with an interstimulus interval of 7 ms. Tone bursts were presented 2000 times, except for intra-operative needle recordings, where 1000 repetitions were sufficient due to the excellent SNR at the place near the cochlea. The word ‘Doris’ (339 ms duration) was used as a speech sample and was presented 500 times at a rate of 2.8 per second. We used condensation and rarefaction polarities.

For the dummy experiments, we selected 0.008 V, as this amplitude elicited responses in the dummy model comparable to those from TM electrodes in humans. The applied voltages were verified with an oscilloscope (TBS 1000C, Tektronix, Beaverton, OR, USA).

During measurements with human subjects, the Eclipse AEP system delivered tone burst stimuli at 65 and 75 dB nHL, corresponding to 89/99 dB peSPL for 500 Hz tone bursts and 94/104 dB SPL for 2000 Hz tone bursts, respectively. The speech stimulus ‘Doris’ was presented at 80 dB nHL, corresponding to 86 dB peSPL. For calibration to normal hearing thresholds, using the Eclipse AEP system, it was matched to a broadband noise with the same frequency distribution and aligned to the level of a 1000 Hz tone. To verify calibration, the insert earphones were connected to an ear simulator (type 4157, Brüel & Kjaer, Nærum, Denmark), and the sound level was measured with a sound level meter (type 2250, Brüel & Kjaer).

Adjusted sound levels in intra-operative measurements approximated those of normal-hearing subjects, considering the individual’s conductive hearing loss. The maximum sound level used was 90 dB nHL for patients with an average hearing loss of around 30 dB HL across mid-frequencies (500 to 2000 Hz).

### 2.8. Signal Processing

This analysis primarily aims to compare the recordings based on their signal-to-noise ratio (SNR), an objective measure, detailed in the subsequent sections. Alongside the SNR, the evaluation of handling, reliability, and flexibility pertains to distinct aspects. Handling and flexibility are partially represented by the characteristics outlined in Table 1 for each device. Flexibility denotes the suitability of these devices for clinical and intra-operative settings, including scenarios involving prolonged stimuli. Reliability involves the capacity of the devices to yield robust results across various conditions for all participants, with even single measurement repetitions providing reasonable data.

The reference AEP system (Eclipse) does not allow full access to raw data, presenting a notable limitation. To ensure better comparability, the analysis was performed on recordings that were averaged over a fixed number of presentations—2000 repetitions for standard stimuli and 500 for speech stimulus—across all devices. This approach was chosen due to the constraints of the AEP system, guiding the selection and adjustment of measurement settings for all evaluated devices. Those recordings acquired with the reference AEP system were exported as an averaged response signal of all measured epochs, with the total number of epochs varying due to online rejection algorithms. The recordings acquired with the alternate devices were imported as a raw continuous signal into MATLAB using EEGLAB [32]. The trigger data and electrode information were checked for errors before each recording was filtered using a 0.5 to 7500 Hz bandpass filter, and epochs were extracted based on trigger time stamps (−1:15 ms; −1:339 ms when using speech stimulus). The epochs were baseline-corrected, checked for extreme amplitude values (>|80 µV|; |200 µV| for needle electrode), and averaged. These steps were taken to address the signal processing of the reference AEP system, including filtering and artifact rejection.

To confirm each device’s frequency response curve, we presented signals of constant amplitude and 1 s duration with an ascending frequency (100 Hz to 10 kHz in steps of 100 Hz) to the dummy model and calculated the power spectrum of the whole recording. The results were normalized to each device’s maximum response power and displayed on a logarithmic scale. For a more detailed comparison, we calculated the area under the curve (AUC) of their respective frequency response curves, using the cutoff frequency f_c_ (−3 dB) as the horizontal axis for comparison. This metric, calculated across the frequency range on the linear scale, represents the total amplification capacity of each device over all frequencies.

For each presented condition, we averaged all repetitions for each participant. From these mean responses, we then calculated the SNR by applying a function adapted from MATLAB’s ‘snr()’, which computes the SNR in decibels relative to the carrier within the frequency spectrum of a sinusoidal input signal. This calculation is based on the energy of the target frequency and its first six harmonics based on a periodogram. However, this method does not account for the non-uniform distribution of EEG noise across frequencies, which may lead to the overestimation of the SNR at higher frequencies. The Fsp method described by Elberling and Don [33], Sininger [34] provides a more robust SNR estimation by considering the variance of the averaged response relative to the variance across epochs. Unfortunately, the Fsp method could not be applied to the Eclipse device data as this device only provides averaged responses and not individual sweeps, which are necessary for this calculation. Subsequently, we conducted statistical comparisons of the calculated SNR values across participants, focusing on the factors of device, electrode type, and stimulus amplitude.

To investigate the relationship between the stimulus and the response signals, we first used cross-correlation to determine the temporal offset between the original speech signal and the response signal. Next, we applied the Hilbert transform to extract the envelope functions of both signals and subsequently low-pass filtered the envelopes using a 6th-order Butterworth filter with a cutoff frequency of 50 Hz. Finally, we computed the Spearman correlation between the stimulus signal envelope and the envelope of the averaged response signal [35]. In the Results Section, we present data from an intra-operative needle recording accordingly.

Some conditions were recorded in alternate polarity (condensation and rarefaction). By subtracting the two averaged responses, the phase-locked CM is emphasized, resulting in an improved SNR [36].

### 2.9. Statistical Analysis

The statistical analysis and data visualization were conducted using RStudio (1 September 2023. Build 494, PBC, Boston, MA, USA) and MATLAB (R2020a, The MathWorks Inc., Natick, MA, USA). We employed a factorial ANOVA to evaluate the influence of various factors on the SNR. This included examining the main effects of devices, electrode types, and stimuli, as well as group effects utilizing Tukey’s honest significance test. The normality of groups was assessed using the Shapiro–Wilk test (shapiro.test() in R), and variance homogeneity was assessed using the Bartlett test (bartlett.test() in R), with no significant irregularities observed. We adapted the procedure for each measurement domain (dummy model, tympanic membrane, intra-operative). This analysis helped determine the most reliable setup for detecting the tested signals, even with a low number of repetitions.

## 3. Results

### 3.1. Dummy Model Measurements

#### Frequency Responses of Amplifiers

**Figure 1:**
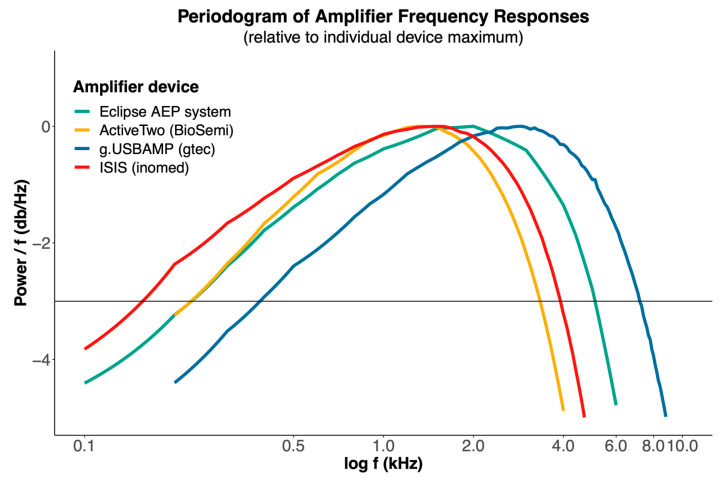
Frequency response curves of the tested devices, demonstrating similarities in frequency-power response with variations in gain and cutoff frequency. Notably, the g.USBAMP device exhibits a slight shift towards higher frequencies. The horizontal line denotes a relative loss of −3 dB of signal power. The different end points of the response curves are a function of each device’s maximal sampling rate.

Our examination revealed distinct frequency response curves for each tested device (Figure 1). We found similar characteristic curves with some differences in gain and cutoff frequency, the latter commonly being identified by a relative decline of −3 dB in signal power. Specifically, the reference AEP system demonstrated a −3 dB threshold of 5.1 kHz and an area under the curve (AUC) of 101.18. The ActiveTwo system showed a lower −3 dB threshold of 3.3 kHz and a reduced AUC of 63.18. The g.USBAMP device had the highest −3 dB threshold (7.1 kHz) and AUC (137.98), while the ISIS system presented a −3 dB threshold of 3.9 kHz and an AUC of 78.53. Lower frequency thresholds can also be derived from the measurements, which indicate different amplification levels of low-frequency stimuli by the respective devices. Those devices that have a lower performance in the high frequency range in turn demonstrate a higher performance in the low frequency range.

### 3.2. Dummy Model Responses

**Figure 2:**
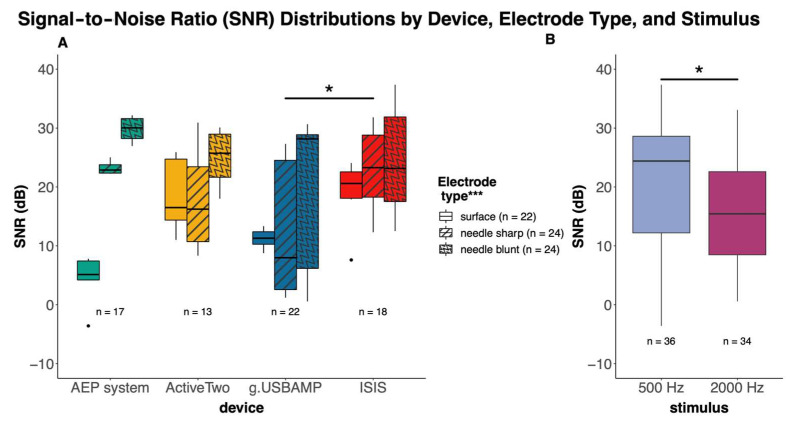
Boxplots displaying the SNR distributions for each device, categorized by electrode type—surface, sharp needle, blunt needle—at the tested frequencies of 500 and 2000 Hz. Differences in the SNR between devices and electrode types are highlighted. Note: Device as a factor has a significant impact (* *p* < 0.5), and the only significant difference is between the g.USBAMP and the ISIS system in individual comparisons. Electrode type as a factor demonstrates a significant influence (*** *p* < 0.001) on the SNR, as indicated. (**A**) Boxplots contrasting SNR values obtained from 500 Hz and 2000 Hz stimuli, illustrating a significant effect (* *p* < 0.5) of stimulus frequency on the SNR across all devices and electrode types (**B**).

Our analysis revealed significant main effects on the SNR by devices (F (3, 6) = 2.8, *p* = 0.04), by electrode types (F (2, 6) = 8.3, *p* < 0.001), and stimuli (F (1, 6) = 5.4, *p* = 0.02), as shown in Figure 2.

Subsequently, post hoc comparisons were conducted to further explore the differences between groups within these factors. Device as a factor indicated a significant difference between ISIS and g.USBAMP (diff = 7.7 dB, 95% CI [0.5, 15.0], *p* = 0.03). Among electrode types, the difference between the blunt needle and surface electrodes was significant (diff = 10.4 dB, 95% CI [4.2, 16.7], *p* < 0.001). For stimuli, a significant difference was observed between the two stimuli 500 and 2000 Hz (diff = 4.9 dB, 95% CI [0.6, 9.1], *p* = 0.03).

### 3.3. Human Measurements

#### 3.3.1. Tympanic Membrane Measurements

**Figure 3:**
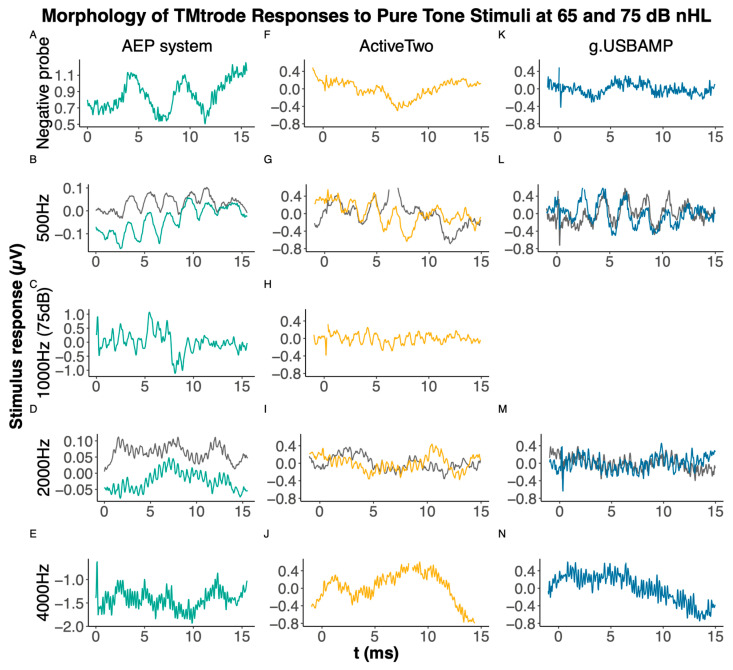
Illustrating the averaged responses from the compared devices for pure tone stimuli at different frequencies at 65 dB nHL (gray lines) and 75 dB nHL (colored lines) using tympanic membrane (TMtrode) electrodes. The sound clamp condition (first row), where the air tubes of the earphones are clamped to test electromagnetic interference, is presented for the Eclipse AEP system (**A**), ActiveTwo (**F**), and g.USBAMP (**K**). Panels (**B**,**G**,**L**) show responses to 500 Hz stimuli, while (**D**,**I**,**M**) display responses to 2000 Hz stimuli. Additionally, test measurements with 1000 Hz stimuli (**C**,**H**) and 4000 Hz stimuli (**E**,**J**,**N**) were conducted at 75 dB nHL. The AEP system does not allow baseline correction as it does not provide access to raw data.

We subsequently assessed TMtrode measurements in normal-hearing subjects, providing a more realistic comparison of the devices. In contrast to the dummy model, we used the Eclipse AEP system as a stimulator to deliver calibrated sound pressure levels. We tested all devices except the ISIS system, due to its technical constraints regarding the trigger that led to a safety issue. As an example, Figure 3 illustrates averaged responses for each device and condition in one subject.

**Figure 4:**
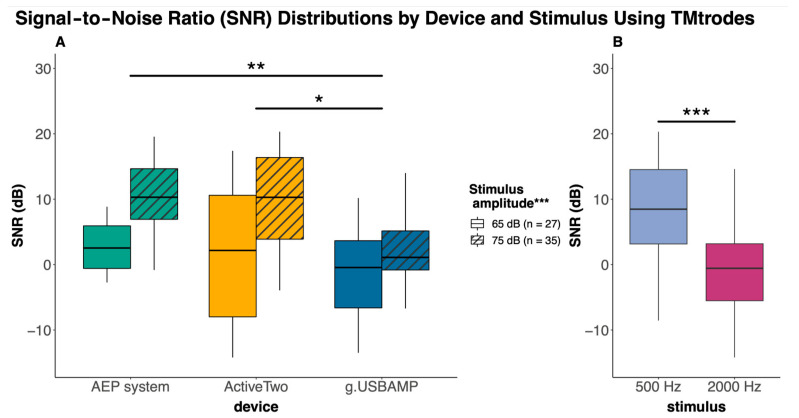
Boxplots comparing SNR values across the Eclipse AEP system, ActiveTwo (BioSemi), and g.USBAMP (gtec) using tympanic membrane electrodes (TMtrode) combining 500 and 2000 Hz stimuli for two different sound levels 65 and 75 dB nHL. Note: Device as a factor had a significant impact (** *p* < 0.01), with significant difference between the g.USBAMP and the Eclipse AEP system (** *p* < 0.01) and between the g.USBAMP and the ActiveTwo system (* *p* < 0.05) in individual comparisons. Stimulus amplitude as a factor demonstrated a significant influence (*** *p* < 0.001) on the SNR, as indicated. (**A**) Boxplots depicting significant (*** *p* < 0.001) SNR difference between 500 and 2000 Hz as stimulus frequency (**B**).

Figure 4 presents boxplots that depict group SNR values comparing the Eclipse AEP system, ActiveTwo (BioSemi), and g.USBAMP (gtec) across sound pressure levels of 65 and 75 dB nHL. We analyzed the averaged responses gathered from all subjects for the tested frequencies of 500 and 2000 Hz.

All main factors exhibited significant effects on the outcome. For the factor device, we observed a significant difference (F (2, 57) = 5.6, *p* = 0.006). As expected, we also observed significantly higher SNR levels when stimuli were presented with a higher sound pressure level (F (1, 57) = 16.7, *p* < 0.001). Moreover, stimulus as a factor had a highly significant impact (F (1, 57) = 32.0, *p* < 0.001).

In our post hoc analysis, we observed significant SNR differences as follows: g.USBAMP consistently exhibited a lower SNR compared to ActiveTwo (−6.4 dB, 95% CI: −11.3 to −1.6, *p* = 0.006) and the Eclipse AEP system (−4.7 dB, 95% CI: −9.2 to −0.2, *p* = 0.04). Additionally, the SNR significantly increased with sound pressure level, showing a 6.5 dB gain at 75 dB over 65 dB across all devices (95% CI: 3.3 to 9.7, *p* < 0.001). Moreover, across devices and measurement conditions, a lower stimulus frequency (500 Hz) was associated with a higher SNR than a higher frequency (2000 Hz), improving by 8.9 dB (95% CI: 5.7 to 12.1, *p* < 0.001).

#### 3.3.2. Intraoperative Trans-Tympanic Measurements

**Figure 5:**
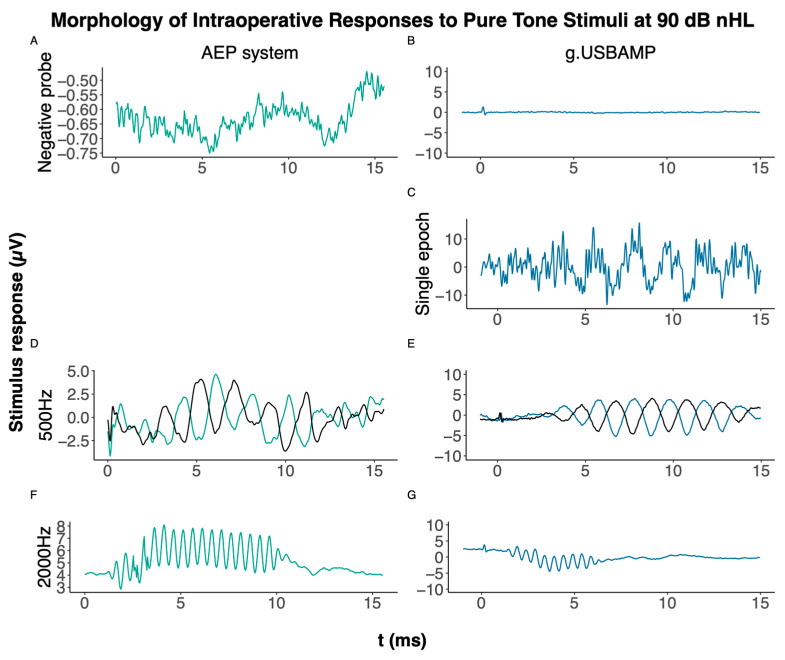
Intraoperative averaged responses of a sound clamp condition, approx. 2000 repetitions —a 10 ms pure tone stimulus at 500 Hz at 90 dB nHL—captured by the Eclipse AEP system (**A**) and the g.USBAMP device, respectively. (**B**) A single stimulus repetition of a 10 ms pure tone stimulus at 500 Hz at 90 dB nHL recorded by the g.USBAMP device, highlighting its capability to capture single epochs. (**C**) A feature not available on the Eclipse AEP system. Responses to both a condensation and a rarefaction pure tone stimulus at 90 dB nHL with pure tone stimulus 500 Hz (**D**,**E**) and 2000 Hz ((**F**,**G**), only rarefaction), approx. 2000 repetitions for both systems. The AEP system does not allow baseline correction as it does not provide access to raw data.

To test the setup, intraoperative measurements via needle electrodes were initially conducted on five patients using both the Eclipse AEP system and ActiveTwo devices. While stimulus responses were successfully recorded with the Eclipse system, no responses were obtained with the ActiveTwo device. Subsequently, measurements were conducted on three patients using the g.USBAMP device. As an example, Figure 5 provides a contrast of intra-operative responses collected by the Eclipse AEP system and the approach presented here utilizing the g.USBAMP device, when subjected to a 10 ms pure tone stimulus at frequencies of 500 Hz and 2000 Hz. The primary focus of this illustration is on the morphology of the response signals rather than their amplitudes. The scales have been adjusted for clarity, but they still differ due to the Eclipse AEP system data not being amenable to post-processing for baseline correction. The acoustic presentation level was individually adjusted for each participant’s hearing deficit. For instance, a participant with a 20 dB conductive hearing loss was effectively stimulated at approximately 70 dB, using a sound level of 90 dB nHL. As proof of principle, we recorded some conditions in both condensation and rarefaction polarity, too.

The sound clamp conditions (A&B) confirmed the absence of any electromagnetic interference that could be associated with the stimulation. Moreover, (C) displays the g.USBAMP device’s recording to a single stimulus repetition, effectively capturing the original 500 Hz pure tone stimulus. The clinical AEP system does not allow for real raw data extraction. Notably, even the data termed as “raw” within the Eclipse AEP system are averaged from multiple repetitions, preventing examination at the level of single epochs.

#### 3.3.3. Speech as Stimulus

To evaluate our setup’s capabilities, we employed a more complex speech stimulus for testing. Utilizing needle electrodes and TMtrodes, we were able to record speech stimuli effectively, with both the ActiveTwo and g.USBAMP device for data capture. While the Eclipse AEP system technically allows for prolonged stimuli, if the stimulus duration exceeds 10 ms, the sampling rate is reduced due to the fixed number of data points per time unit, significantly compromising signal resolution.

**Figure 6:**
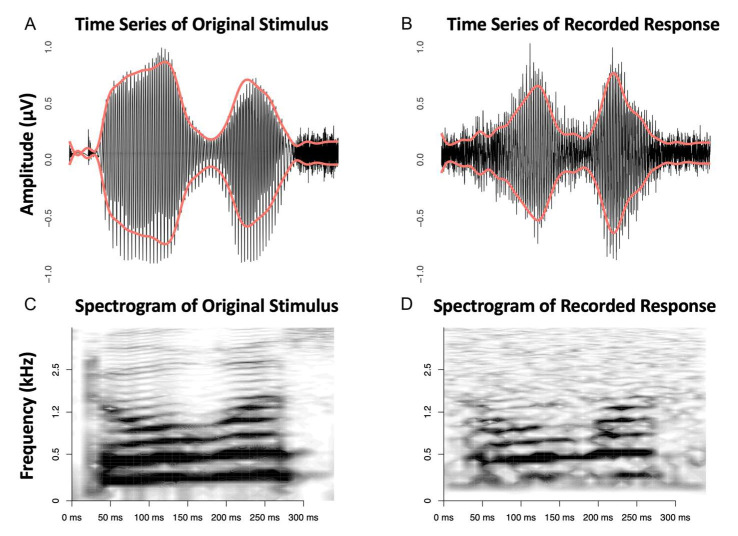
Time series of the original stimulus ‘Doris’ (**A**) and the recorded response (**B**) using the g.USBAMP device, with red overlays indicating the waveform envelopes. The corresponding spectrograms (**C**,**D**), reveal the frequency content over time. The left set illustrates the original stimulus of 339 ms, while the right set demonstrates the processed response signal, obtained by subtracting the average responses to condensation and rarefaction, presented at alternate polarity, and repeated 500 times. The red lines demonstrate the lowpass-filtered envelopes of the signals.

As an example, Figure 6 shows an intraoperative recording captured via a needle electrode with the g.USBAMP. The response signal exhibits a remarkable similarity to the original stimulus, both in the time domain and in the spectrogram. This is particularly evident in the fundamental frequency range around 100–200 Hz and a more prominent signal component around 500 Hz. High-frequency signal components that are evident in the original are also preserved recognizably in the cochlear microphonic response.

Upon converting the stimulus response into a wave file for playback, the original stimulus was distinctly audible, and the voice was identifiable (audio samples can be found in the Supplementary Materials). For a more quantitative assessment of the resemblance between the stimulus and the response, we correlated the lowpass-filtered envelopes of both signals. The Pearson correlation coefficient I was found to be 0.78, indicating a strong positive correlation.

## 4. Discussion

Our study employed laboratory measurements, recordings from healthy participants, and intraoperative patient recordings to assess various signal recording devices in comparison with a clinical AEP system, focusing on cochlear microphonics (CMs). The findings of this study highlight the potential of the EEG amplifiers tested in the proposed setup, revealing their strengths and weaknesses, and providing key insights for future research and clinical applications.

The primary goal of our analysis was to compare recordings based on their SNR, an objective measure we thoroughly detailed in subsequent sections. In addition to the SNR, we evaluated handling, reliability, and flexibility, which are distinct yet crucial properties for the overall performance. Handling and flexibility are partially captured by the features detailed in Table 1 for each device. Flexibility specifically refers to the devices’ adaptability for use in both clinical, intra-operative and research settings, which includes the capability to handle prolonged stimuli. Our analysis found that certain devices were unsuitable for intra-operative use, underscoring the diverse capabilities and constraints revealed during our evaluation.

**Technical review**

Selecting the appropriate signal amplifier for our experiments was guided by several key criteria: an adequate sampling rate of at least 20 kHz, a frequency response that is as uniform as possible across the 100 to 8000 Hz range, and compatibility with passive electrodes in combination with TMtrodes or blunt needle electrodes. We employed a trigger box to ensure the precise synchronization of recordings, enabling the accurate alignment of repeated measurements with stimulus presentation. Despite minor deviations of ± 1 sample, this method proved largely accurate, given a sufficiently high sampling rate that mitigated the impact of such deviations.

The reference AEP system, which is a clinical tool frequently used for measuring cochlear potentials and brainstem responses to auditory stimuli, offers advantages such as the perfect synchronization of recorded epochs and a preamplifier system that considerably reduces interference in the connection cable to the amplifier. However, the system, while optimized for certain tasks, is limited in that it cannot handle tone bursts longer than 15 ms due to a capacity of 467 data points per epoch, thus constraining the duration of sound presentation and rendering it unsuitable for certain scientific inquiries. Many studies rely on AEP systems, sharing similar technical limitations, particularly regarding the limited number of data points per epoch, which directly affects both the recording window length and the feasibility of presenting extended sound stimuli. This constraint can result in either a limited recording window length or a suboptimal sampling rate, potentially impacting data quality and detail. For instance, Riggs, Hiss [19] used a Biologic Navigator Pro AEP system with a maximum of 1024 sampling points per epoch, translating to a sampling rate of about 10 kHz at a 100 ms epoch length. In contrast, by using an external sound interface with an EEG system that supports continuous data collection, our approach enhances the presentation of prolonged stimuli, demonstrating a notable advancement in capability. An alternative to extending the window length could be using a low-frequency signal and its harmonics to span the human auditory frequency range.

Simpson, Jennings [37] described other research equipment (TDT, Alachua, FL, USA) for measuring cochlear potentials. However, their study was confined to click stimuli using tympanic membrane electrodes and did not encompass intraoperative needle measurements or tone bursts as stimuli. This system, akin to our clinical AEP system, demonstrated the optimal synchronization of recorded stimulus responses due to its integrated components, making it a suitable choice. Nonetheless, it was not used in our study due to a lack of the requisite safety approvals for human studies within our operational framework. Furthermore, Kumaragamage, Lithgow [38] employed another suitable signal amplifier (CED 1902, Cambridge Electronic Design Limited, Cambridge, England), which, unfortunately, is also not available for the EU market.

The elevated baseline noise in our new setup can be attributed to several factors: The conventional AEP system has optimized hardware filters and rejection algorithms for specific applications, unlike alternative devices. The g.USBAMP device captures a larger signal band due to its higher sampling rate and broader frequency, thereby recording more noise. Pre-amplification in the AEP system and active electrode use in the ActiveTwo device both help reduce interference. We repeatedly faced interference and grounding issues. There were significant interference signals from each stimulation device used, even when an appropriate distance was maintained.

The ActiveTwo device performed well in measurements with the surface and TMtrodes, in terms of the SNR, on par with the clinical AEP system. However, despite repeated attempts, we were unable to reliably record responses to stimuli in the intraoperative setting using needle electrodes. A plausible cause could be the device’s different approach to referencing, employing a Common Mode Sense (CMS) active electrode and Driven Right Leg (DRL) passive electrode for noise suppression. This technique might interact adversely with other clinical monitoring systems like anesthesiology equipment, potentially leading to inconsistent data. While clinical systems like the g.USBAMP offer selectable low- and high-pass filters, allowing for a wider frequency range, the complexity of research systems may limit their use in clinical settings. Progress in this area may be achieved by developing a unified device that integrates both the recorder and stimulator, reducing noise interference and better meeting clinical recording demands.

**Dummy model**

Amplifiers demonstrated distinct frequency response variations in our tests. Specifically, the g.USBAMP device exhibited the maximum lowpass cutoff frequency and the widest frequency detection range, also expressed by the greatest AUC, which is vital given the role of high fidelity and signal power in the higher frequency domain for processing human speech signals [39,40]. The AEP system, designed for recording cochlear and brainstem potentials in response to acoustic stimuli, captures a broad range of frequencies, from low (<500 Hz) to relatively high frequencies (up to a 5.1 kHz −3 dB cut off). In contrast, the alternative systems, ISIS and ActiveTwo, demonstrated obviously more constrained frequency responses. In particular, the ActiveTwo devices’ performance in the high-frequency range was restricted due to its lower sampling rate of 16 kHz. Consequently, our findings highlight the superior sensitivity of the g.USBAMP device in the higher frequency band and its broad frequency range signal detection.

Given the AEP system’s restrictions on maximum stimulus duration for tone bursts and the fixed number of data points per recorded epoch, we adopted a uniform 10 ms time window for most conditions across all devices. Traces termed “raw data” in the AEP system were in fact averages of 10 repetitions, limiting the examination of individual stimulus repetitions and detailed analysis as in Figure 5. Unlike the AEP system, the alternative devices allowed a retrospective review of an arbitrary number of averaged epochs, aiding a more detailed examination of the recording’s sensitivity and the temporal relationship between stimulus and response.

For comparative analyses, we utilized surface electrodes, which revealed the most significant differences among the devices. The g.USBAMP showed significantly lower SNR values compared to both the ISIS and the Eclipse AEP system. This disparity is likely attributable to the latter systems’ superior optimization for clinical monitoring in the noise-intensive environments of operating rooms. The blunt needle electrode consistently offered a better SNR, whereas surface and sharp needle electrodes showed relatively higher impedance levels. This reflects expectations, as the blunt needle, with its larger pin surface and needle electrodes, in general, can reach closer to the signal source, improving signal reception and quality. Earlier, Jünemann, Hoth [41] proposed a passive patient simulator to replicate the geometric and electrical properties of scalp electrode placement, providing a standardized testing approach. However, their solution, based on a resistor–capacitor network on a circuit board, appears to offer limited flexibility for testing different electrode types, as achieved with our dummy model.

**TMtrode in healthy humans**

Our approach, which proved to be effective in highlighting differences between devices in the melon model measurements, was subsequently applied to measurements with human participants. Although the g.USBAMP device demonstrated a lower SNR compared to the ActiveTwo device and the reference AEP system, it showed comparable performance at the higher 2000 Hz stimulation frequency, underscoring its proficiency in handling higher frequencies. Consistent with Zhang [12], increasing the stimulus amplitude (to 75 dB) significantly improved the SNR uniformly across all devices. These findings essentially align with frequency response curves: the AEP system excels at lower frequencies, while the g.USBAMP shows advantages at higher frequencies. We infer that the technical benefits of these devices (AEP system, ActiveTwo) are particularly pronounced in practical measurement contexts, given their design for optimal performance in such environments. Although the g.USBAMP device may seem technically simpler and less sophisticated, it nevertheless consistently delivers robust and reliable results, proving its effectiveness even in intraoperative settings. The disparities in the SNR and electrode types visible in between measurements within the dummy model and tympanic membrane measurements in human subjects underscore the technical nuances that can influence data quality.

**Intraoperative speech recordings**

During initial intraoperative needle measurements using a blunt needle and the ActiveTwo device, desired results were not achieved in five subjects. Subsequently, the g.USBAMP device was employed, producing favorable responses in three consecutive measurements, comparable to the reference AEP system. The impedance of needle electrodes was considerably higher than that of surface electrodes and TMtrodes.

The g.USBAMP device consistently and reliably recorded stimulus responses across the different modalities. It captures the original pure tone stimulus in a single repetition, as illustrated in Figure 5C—a depth of raw data analysis unattainable with the AEP system. This comparison is qualitative in nature, not derived from statistical analysis.

Visible spikes at the beginning of the recorded responses for the ActiveTwo and g.USBAMP devices can be observed in Figure 3 and Figure 5. These are most likely negligible electromagnetic interferences due to the comparably strong external trigger signal (5V TTL) transmitted to the amplifier via the trigger box. Nevertheless, a higher baseline noise is observed with g.USBAMP due to its increased sampling frequency. In a sound clamp condition, where no stimulus was presented by clamping the tubes, none of the devices registered stimulation artifacts, confirming the absence of false positives due to electrical crosstalk.

In a final step, we employed an intra-operative needle recording to capture the biological response to a short speech stimulus presented in alternating polarity. We found a distinct audible quality of the recording and high correlation with the original stimulus, thus confirming success in our goal to describe a promising setup for electrocochleography research.

## 5. Conclusions

The present study suggests a suitable method for recording cochlear microphonics using standard EEG amplifiers. This allows extended measurement windows to be realized at high sampling rates, which is a clear improvement over the clinical AEP systems. This approach can be used to investigate cochlear responses to complex stimuli or to perform correlation analyses between continuous speech signals and the cochlear signals elicited by them.

## Figures and Tables

**Table 1:** Detailed specifications of the evaluated amplifier devices. Beyond different levels of suitability, the systems vary in their maximum sampling rates, window lengths, and −3 dB cutoff frequencies. The latter were determined in this study. Another version of the g.USBAMP with a −3 dB cutoff frequency of 18.8 kHz is available.

	Eclipse AEP System (Interacoustics)	ActiveTwo (BioSemi)	g.USBAMP (gtec)	ISIS (Inomed)
Max. sampling rate (kHz)	30 or conditional to recording window length.	16	38.4	20
−3 dB cutoff (kHz)	7.5	3.2	~18.8/7	5
Max. recording window length.	Depends on sampling rate: 30 kHz allows for 15 ms, 15 kHz allows for 30 ms, and 3 kHz allows for 150 ms.	Unlimited recording duration.		
On-board filter (Hz)	Hardware high-pass filter: 0.5 Low-pass filter: 7500	High-/low-pass filter: optional	High-/low-pass filter: optional	Hardware high-pass filter: 0.5 Low-pass filter: optional
Trigger	Integrated, analog in/out	Digital input (SPDIF)	Analog/digital input	Analog input
Mobility	For clinical use.	Stationary use in research lab.	Compact system for research and medical use.	For clinical use.
Advantages	- Safe handling - Calibrated system - Clinically reliable and controlled measurements	- 16 channels - High reproducibility with TMtrode - Suitable for research	- 16 channels - Certified medical device - Real-time data embedding - High sampling rate	- 8 channels - Certified medical device - High sampling rate
Disadvantages	- Limited configurability - Short recording duration (15 ms) - No raw data access	- Not certified for clinical use - Not suitable for intraoperative measurements - Low sampling rate	- Not applicable for clinical diagnostics	- Technical safety limitations prevent human ECochG measurements

## Data Availability

The data presented in this study are available upon reasonable request from the corresponding author.

## Supplementary Materials

The following supporting information can be downloaded at https://www.mdpi.com/article/10.3390/audiolres15010008/s1, Audio S1: Doris stimulus, Audio S2: Doris response, Table S1: Electrodes and Placements, Figure S1: Triggerbox_circuit_diagram.
