# Supplementary material for: High-Resolution EEG Amplifiers Are Feasible for Electrocochleography Without Time Restriction
Source: Audiol Res. 2025 Jan 21;15(1):8. doi: 10.3390/audiolres15010008 (PMC11851963; doi:10.3390/audiolres15010008)

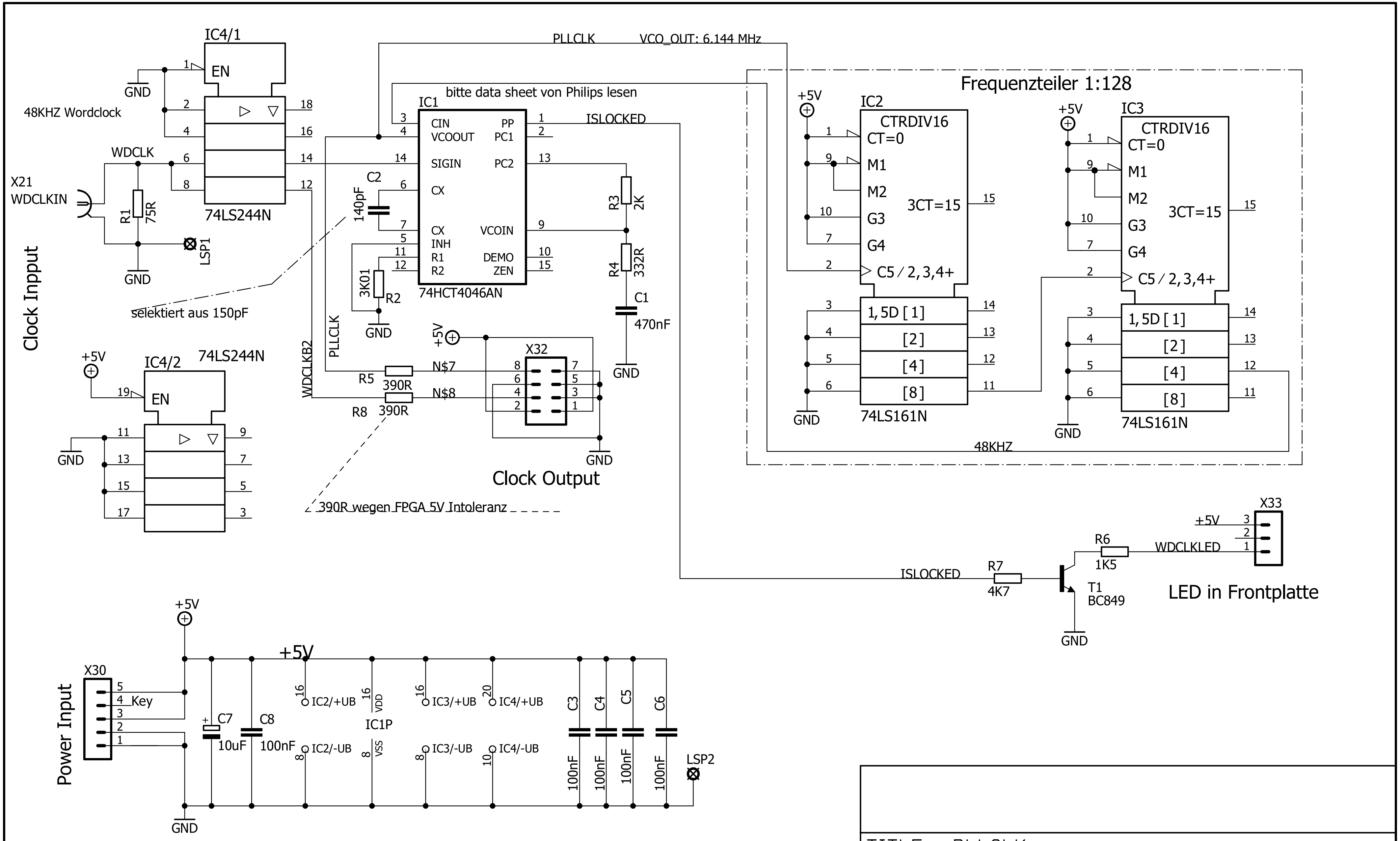

TITLE: PLLCLK

TTL Triggerbox

Document Number: 123 / 2021

REV:

Date: nicht gespeichert!

Sheet: 1/1

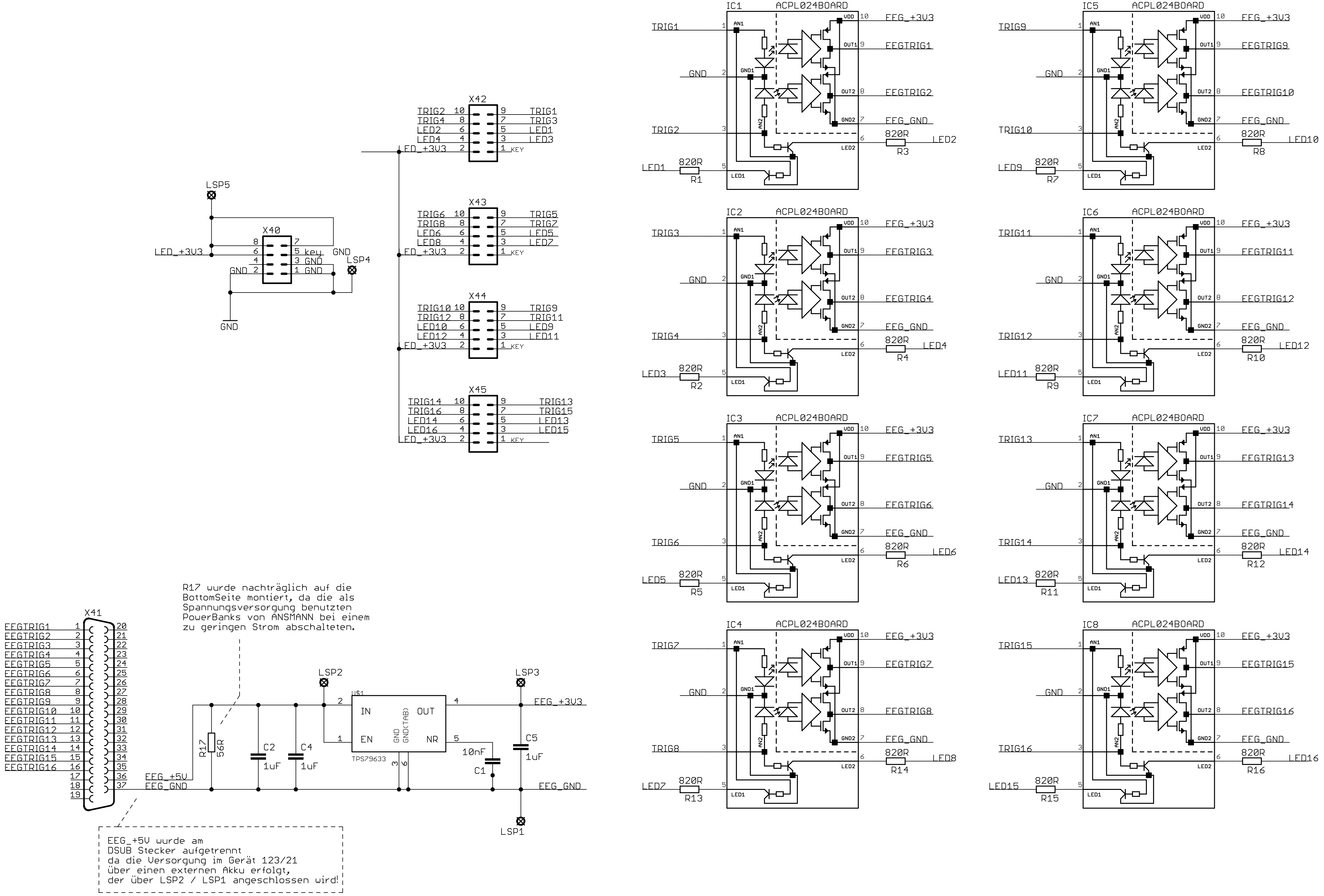

X41
EEGTRIG1
EEGTRIG2
EEGTRIG3
EEGTRIG4
EEGTRIG5
EEGTRIG6
EEGTRIG7
EEGTRIG8
EEGTRIG9
EEGTRIG10
EEGTRIG11
EEGTRIG12
EEGTRIG13
EEGTRIG14
EEGTRIG15
EEGTRIG16
EEG_+5V
EEG_GND
R17 wurde nachträglich auf die BottomSeite montiert, da die als Spannungsversorgung benutzten PowerBanks von ANSMANN bei einem zu geringen Strom abschalteten.
R17
56R
C2
1uF
C4
1uF
LSP2
IN
EN
OUT
NR
GND
GND(TAB)
TPS79633
10nF
C1
C5
1uF
LSP3
EEG_+3V3
EEG_GND
LSP1
EEG_+5V wurde am
DSUB Stecker aufgetrennt
da die Versorgung im Gerät 123/21
über einen externen Akku erfolgt,
der über LSP2 / LSP1 angeschlossen wird!
LSP5
X40
LED_+3V3
key
GND
LSP4
X42
X43
X44
X45
TRIG1
TRIG2
TRIG3
TRIG4
LED1
LED2
LED3
LED4
LED_+3V3
KEY
IC1
ACPL024BOARD
820R
R1
R3
EEG_+3V3
EEGTRIG1
EEGTRIG2
EEG_GND
IC2
IC3
IC4
IC5
IC6
IC7
IC8

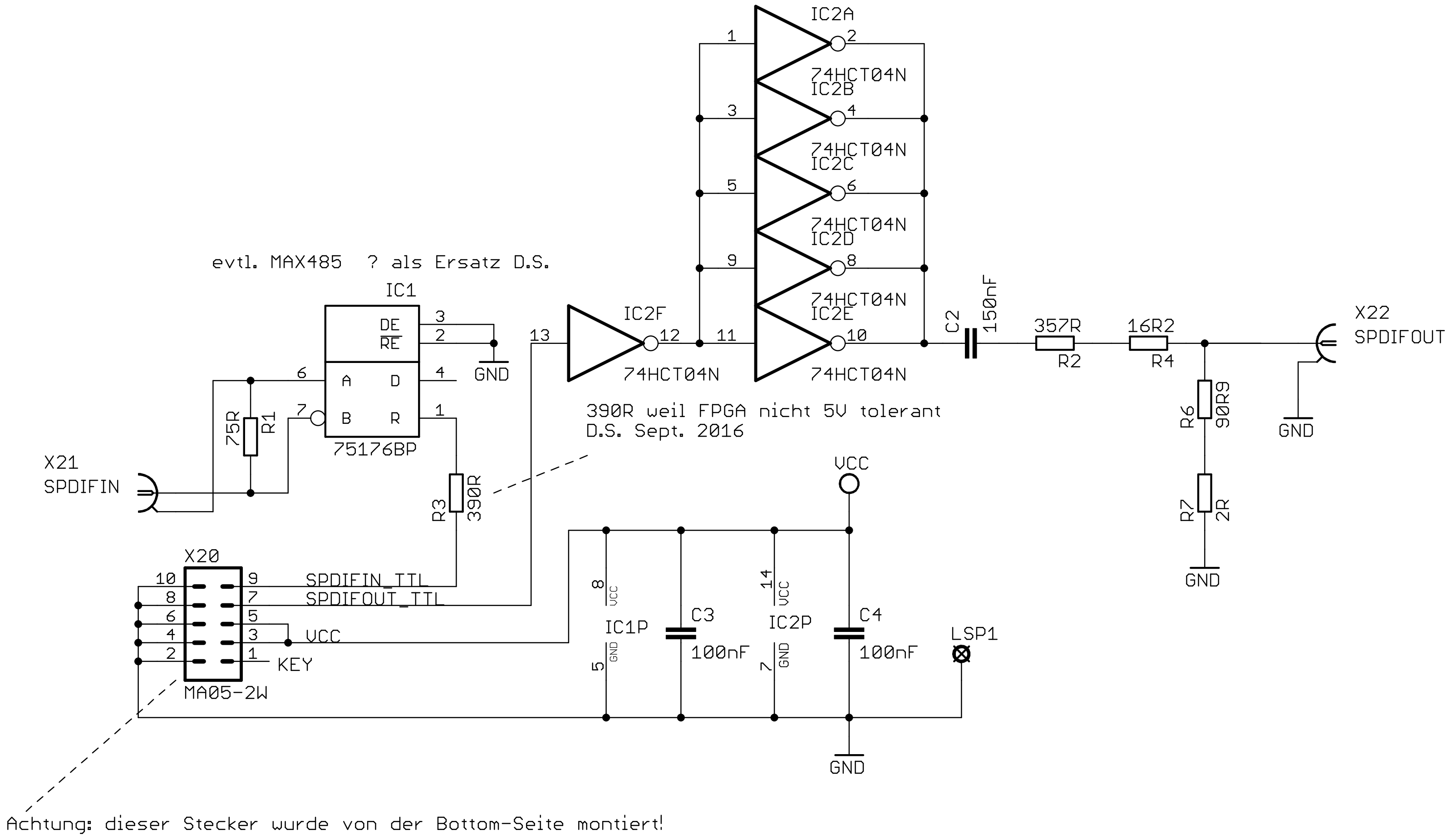

123 / 2021
SPDIF Interface
TTL Triggerbox

SPDIF_INOUT
09.03.2021 11:10
Sheet: 1/1

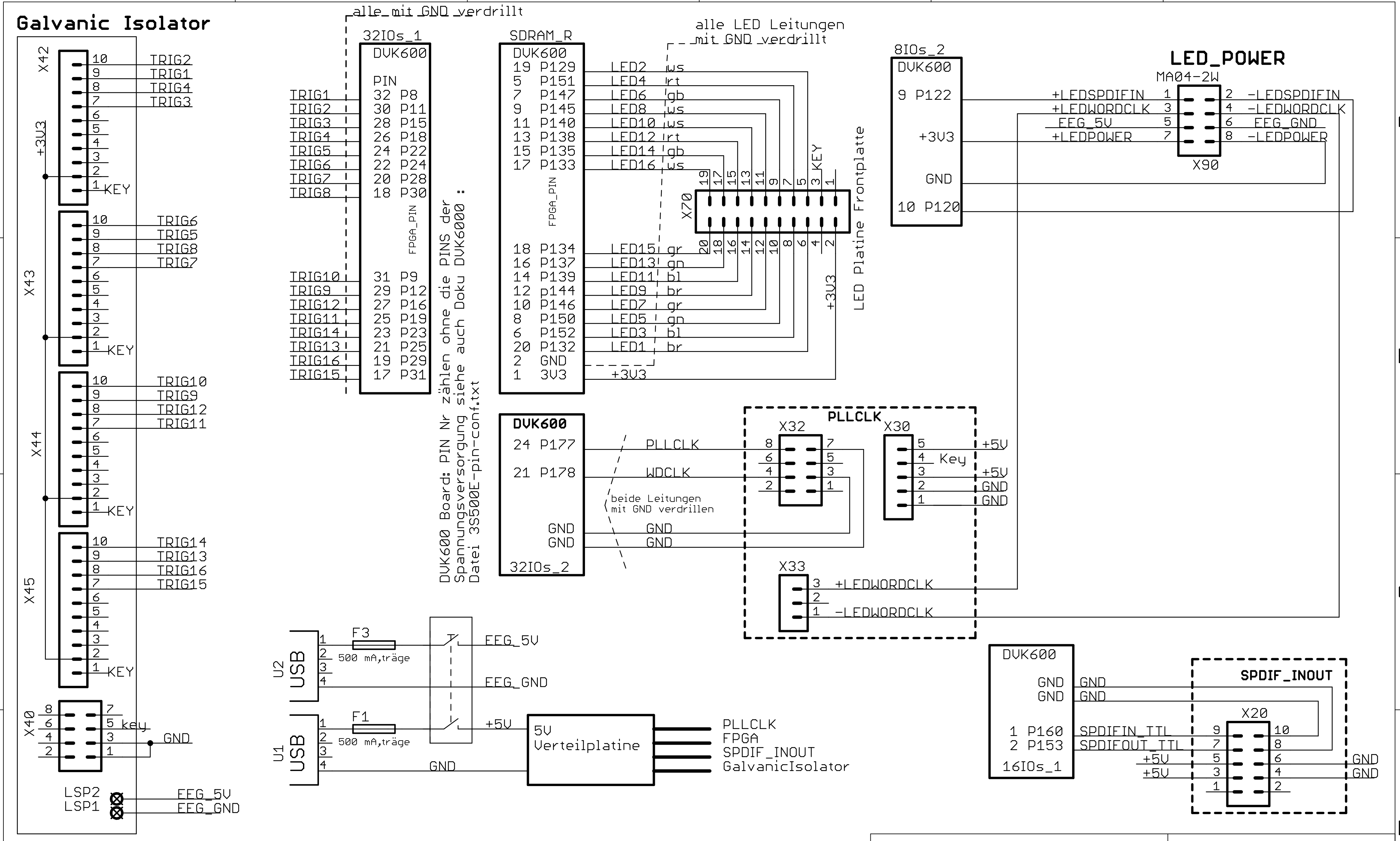

Diese Triggerbox wird über zwei Ansmann Powerbanks 8.0 mit zwei 5V Spannungen versorgt.

123 / 2021

Trigger Box

Verdrahtung

09.01.2024 17:26

Sheet: 1/1

der 6.144MHz Takt wird von der PLL-
Schaltung 74HCT4046 generiert.

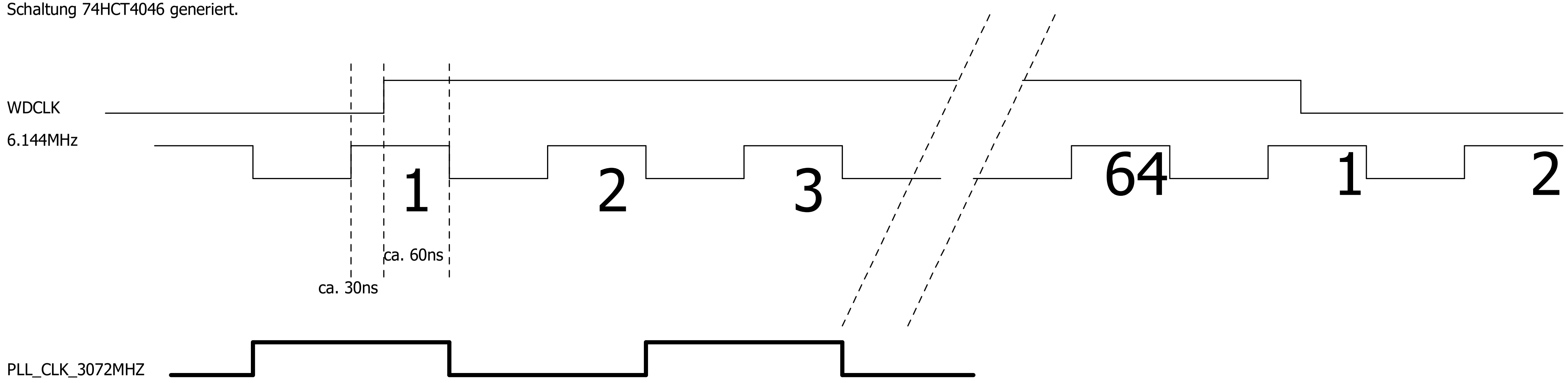

Supplement: Supplementary file 1 [file audiolres-15-00008-s001.zip › 2024_Schertenleib_supplemental_materials/2024_Schertenleib_Triggerbox_circuit_diagram.pdf]
