# Supplementary material for: High-Resolution EEG Amplifiers Are Feasible for Electrocochleography Without Time Restriction
Source: Audiol Res. 2025 Jan 21;15(1):8. doi: 10.3390/audiolres15010008 (PMC11851963; doi:10.3390/audiolres15010008)
Supplement: Supplementary file 1 [file audiolres-15-00008-s001.zip › 2024_Schertenleib_supplemental_materials/2024_Schertenleib_Electrodes_And_Placements.pdf]

Electrodes

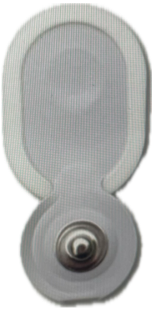

Surface electrode: We used Ambu Neuroline 720 surface electrodes (Ambu GmbH, Germany), which were attached to the skin after superficial cleaning and application of conductive gel.

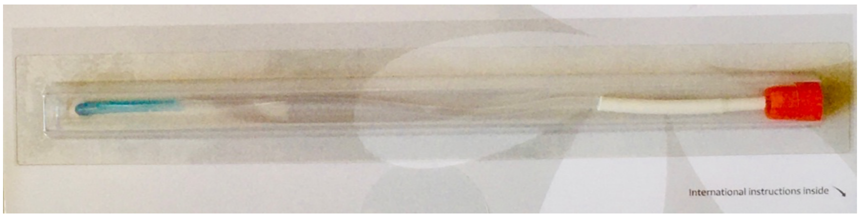

Tympanic membrane electrode (TMtrodes): A plastic-coated wire with a safety tip covered in gel, which are placed in the ear canal proximal to the tympanic membrane in the inferior region and fixed by placing the earphone tip in the ear canal. In this study we used TM electrodes for ECochG by Sanibel™ (USA).

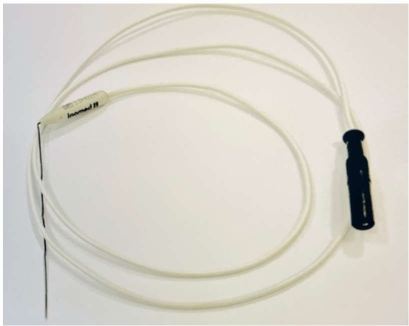

Needle electrode: The needle is usually sharp at the tip to better incise the eardrum. It can also be blunt to provide a better contact surface and thus better conduction. Needle measurements are usually taken intraoperatively. Patients must lie still, as needle placement requires absolute rest. The needle usually is placed near the round window niche and then like the TMtrode, fixed in position by placing the earphone tip in the ear canal. In our setup we used both sharp and blunt needle electrodes for ECochG by inomed Medizintechnik GmbH (Germany).

Placements

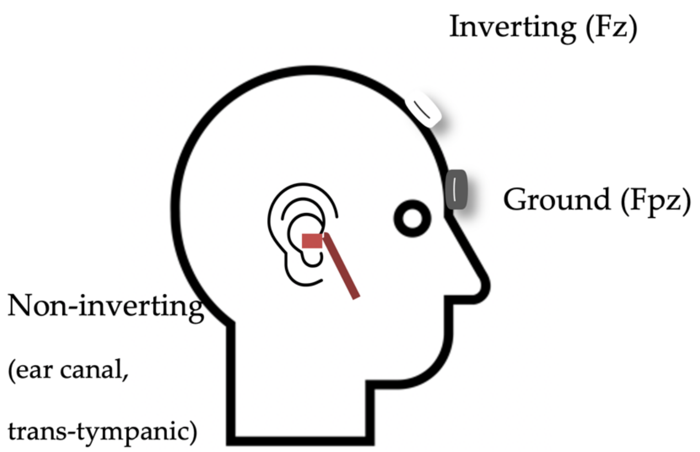

Electrode placement in participant measurements: In our setup, we followed the instructions of the manufacturer of the Eclipse AEP system and placed the inverting electrode on the forehead (Fz). This electrode setup was applied to all measurements in this study.

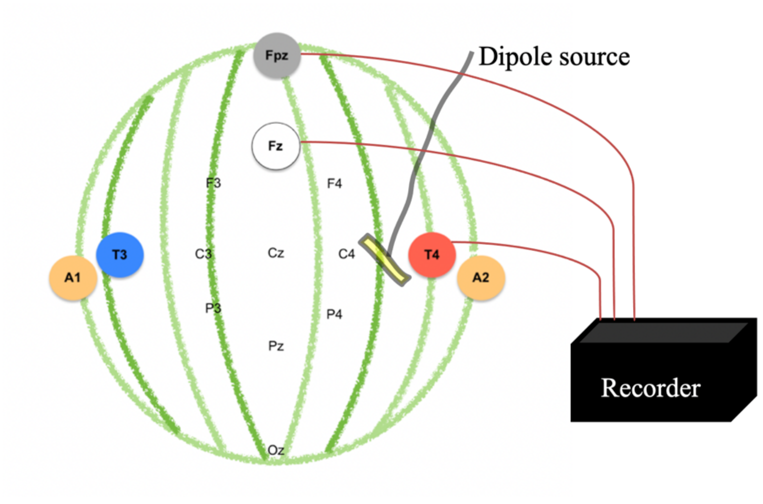

Electrode placement in dummy model: After fixating the dipole source in its place, surface- and needle electrodes were attached according to the 10-20 electrode system and fixated too. A ground electrode was placed on Fpz, inverting (reference) on Fz, non-inverting surface electrode and needle electrodes (sharp and blunt) were placed on one temporal pole, approximately on A1/T3 or A2/T4 respectively.
